# Role of Focal Adhesion Kinase and NRF2 Pathways in Gastrointestinal Wound Healing

**DOI:** 10.3390/ijms27146335

**Published:** 2026-07-16

**Authors:** Olivia G. Cleveland, Emilie E. Vomhof-DeKrey

**Affiliations:** 1 Department of Pathology, School of Medicine and the Health Sciences, University of North Dakota, Grand Forks, ND 58201, USA; olivia.cleveland@und.edu; 2 Department of Biomedical Sciences, School of Medicine and the Health Sciences, University of North Dakota, Grand Forks, ND 58201, USA; 3 Department of Surgery, School of Medicine and the Health Sciences, University of North Dakota, Grand Forks, ND 58201, USA

**Keywords:** epithelial restitution, reactive oxygen species, antioxidant, mucosal repair

## Abstract

The gastrointestinal epithelium forms a critical barrier that regulates nutrient absorption while preventing the translocation of harmful luminal contents. Disruption of this barrier initiates a coordinated wound healing response involving epithelial restitution, proliferation, and differentiation. Focal adhesion kinase (FAK) is central to this process, regulating focal adhesion (FA) turnover and cytoskeletal dynamics required for epithelial migration. Activation of FAK via phosphorylation at tyrosine 397 (Y397) promotes cell motility, proliferation, and survival, whereas loss of function impairs mucosal repair and exacerbates tissue injury. Effective wound healing also requires tight regulation of reactive oxygen species (ROS). The nuclear factor erythroid 2-related factor 2 (NRF2) pathway governs antioxidant defenses by inducing cytoprotective genes, including SOD1, CAT, GCLC, GCLM, and NQO1, restoring redox homeostasis and limiting inflammation. NRF2 deficiency results in increased oxidative stress, heightened inflammatory signaling, and delayed wound healing. While both FAK and NRF2 are independently essential for gastrointestinal wound healing, their mechanistic relationship remains unclear. Emerging evidence suggests that they may be functionally linked through redox-dependent signaling where FAK-mediated ROS production may promote NRF2 activation, while NRF2-driven antioxidant responses maintain conditions necessary for sustained FAK signaling. This coordinated interaction highlights a redox-sensitive feedback mechanism critical for efficient gastrointestinal wound repair.

## 1. Introduction

Gastrointestinal wound healing is essential for maintaining epithelial barrier integrity and protecting the body from inflammation, infection, or chronic gastrointestinal diseases. Disruption of this repair process plays an important role in disorders such as inflammatory bowel disease, chronic inflammation, and colorectal cancer. While FAK signaling and NRF2-mediated antioxidant responses are each independently known to regulate epithelial repair, there is increasing evidence suggesting that these pathways may function collaboratively through redox-sensitive signaling mechanisms during wound healing. The goal of this review is to summarize current understanding of the roles of FAK signaling, NRF2-mediated antioxidant regulation, and downstream antioxidant genes in intestinal wound healing while highlighting important knowledge gaps and presenting a conceptual framework to guide future studies investigating FAK-ROS-NRF2 crosstalk in this process. Understanding how FAK and NRF2 coordinate during epithelial healing may identify novel therapeutic targets for diseases characterized by impaired mucosal healing and chronic oxidative stress.

## 2. Gastrointestinal Wound Healing

The gastrointestinal epithelium provides a selectively permeable barrier that separates luminal contents from underlying tissues while performing two key physiological functions. First, it facilitates and regulates the absorption of nutrients into the body and, secondly, it identifies and intercepts harmful molecules and microorganisms, inhibiting their passage further into the body [1]. Maintenance of this barrier is critical for intestinal homeostasis and is dependent on a dynamic balance between mucosal injury by destructive factors and healing via protective factors [2]. A key feature supporting this balance is the rapid turnover of gastrointestinal epithelial tissue, with complete turnover of most cells occurring every 3–4 days [3]. This continuous renewal of the epithelium enables replacement of damaged or stressed cells while preserving barrier integrity despite the continuous exposure to luminal stressors. These stressors range from mechanical abrasion from luminal contents to excessive gastric acid or pepsinogen secretion, digestive enzymes, ethanol consumption, and medications (nonsteroidal anti-inflammatory drugs (NSAIDs)). Injury also occurs in disease states when there is substantial inflammation caused by inflammatory bowel diseases or during bacterial infections, like *Helicobacter pylori* [2,4].

Under healthy physiological conditions, repair mechanisms efficiently counterbalance injury maintaining a dynamic equilibrium. However, this balance can be easily disrupted by excessive or sustained damage resulting in compromised gastric barrier integrity. When disruption occurs, luminal microbes and toxins can penetrate deeper into the mucosa, triggering tissue injury and inflammatory responses [5]. To restore barrier function, the gastrointestinal mucosa initiates a highly coordinated wound healing response dependent on the restitution, proliferation, and differentiation of epithelial cells adjacent to the wounded area [6]. Although these three processes are often described as distinct phases, it is important to note that they merge into one another and overlap in vivo [7].

**Figure 1:**
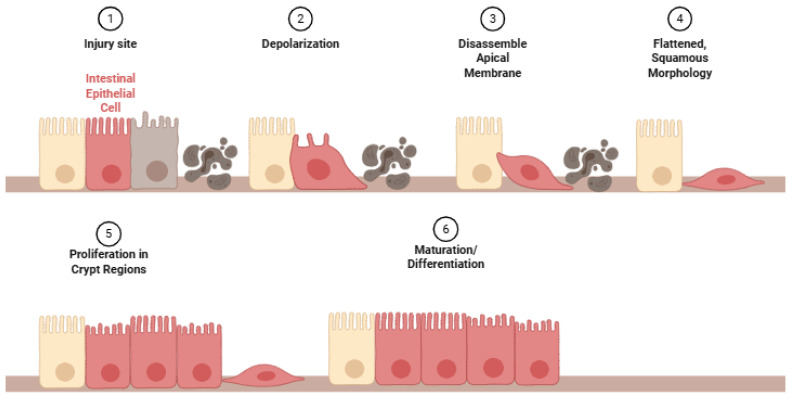
Epithelial restitution phases of mucosal repair followed by proliferation and maturation at the injury site.

Epithelial restitution represents one of the earliest phases of mucosal repair and is independent of cell proliferation [8]. Within minutes to hours of injury [8], epithelial cells or absorptive enterocytes [9] near the wound site depolarize and lose their typical morphology, completely disassemble their apical specialized membrane components [10], and adopt a flattened, squamous morphology (Figure 1). These cells extend lamellipodia and pseudopodia-like structures to migrate across the denuded basement membrane, where they subsequently repolarize to restore barrier integrity over the exposed basement membrane [2].

If the wounded area is extensive enough, epithelial cell proliferation increases, primarily in the crypt regions containing stem cells and the transit-amplifying progenitor cells [11], behind the migrating epithelial sheet to provide enough cells to migrate across and cover the wound [2]. Subsequently, maturation and differentiation of newly formed undifferentiated epithelial cells to differentiated cells such as goblet and paneth cells [12,13] are necessary to maintain the functional architecture of the epithelium [8]. Extensive injuries would also need migration and proliferation of fibroblasts [14], endothelial cells [15], and smooth muscle cells [16].

## 3. FAK Signaling Pathway in Intestinal Wound Healing

During epithelial restitution, cells adjacent to the wounded area flatten and migrate into the wounded area to reseal the epithelial barrier by extending lamellipodia that are stabilized to the underlying matrix via focal adhesion (FA). This process depends on dynamic remodeling of FAs, which provides traction forces necessary for cell movement. FAK regulates FA turnover, enabling efficient epithelial migration. As a key mediator of integrin- and growth factor-dependent signaling, FAK integrates extracellular matrix (ECM) cues with intracellular pathways that control epithelial migration, focal adhesion turnover, proliferation, and survival processes that are essential for effective mucosal repair following injury [17,18,19].

**Figure 2:**
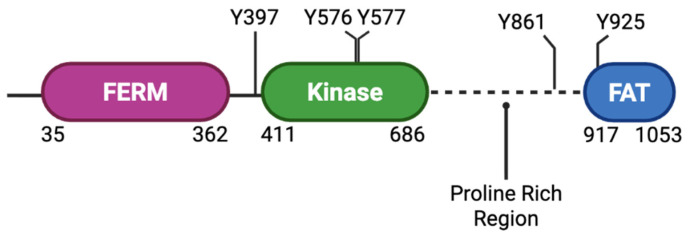
FAK domains and tyrosine phosphorylation sites.

Structurally, FAK contains an N-terminal FERM domain, a centrally located catalytic tyrosine kinase domain, a C-terminal focal-adhesion targeting (FAT) domain (a four-helix bundle), and an unstructured proline-rich region between the catalytic and FAT domains (Figure 2) [14]. Upon integrin engagement with the ECM, FAK is recruited to FAs and undergoes autophosphorylation at Y397, creating a docking site for Src family kinases and initiating downstream signaling cascades that regulate cytoskeletal dynamics and cell motility [14,20,21]. Phosphorylation of Y397 is critical for most biological activities controlled by FAK, as shown with a Y397F-mutated mouse model where the tyrosine is not able to be phosphorylated, resulting in embryonic lethality [22].

Following injury, FAK expression and phosphorylation normally increase to drive repair. Interestingly, at the ulcer or injury margins, FAK protein and phosphorylated FAK are reduced before being induced in the adjacent repairing epithelium. This indicates that restoring FAK signaling is part of the healing response [23].

Phosphorylation of FAK and its interaction with the Src protein promote the assembly and disassembly of adhesion complexes, enabling a coordinated forward movement of epithelial cells [24]. In the absence of FAK, cells exhibit enlarged, stable adhesions and impaired migratory capacity, highlighting its importance in adhesion turnover and efficient wound closure [25,26]. Consistent with this, inhibition or suppression of FAK activity significantly slows epithelial migration and delays wound healing in in vitro models [27]. In *H. pylori* infections where the pilus-associated virulence factor cytotoxin-associated gene A (CagA) is phosphorylated, this causes a reduction in FAK phosphorylation through the recruitment of the Src homology region 2 domain containing phosphatase-2 (SHP-2) [4,28]. Similarly, the *H. pylori* secreted virulence factor vacuolating cytotoxin A (VacA) also reduces FAK activity [4,29].

FAK coordinates signaling networks that link FA dynamics to cytoskeletal reorganization. Through interactions with adaptor proteins such as paxillin, FAK facilitates the recruitment of signaling molecules that regulate actin remodeling and directional migration. Effective cell motility involves phosphorylation of paxillin by FAK which promotes the assembly of protein complexes with nascent adhesions at the leading edge of the cell followed by disassembly at the trailing edge [30]. Disruption of FAK–paxillin interactions impairs adhesion signaling and reduces cell migration and invasion, underscoring the importance of this axis in epithelial repair [31].

Beyond migration, FAK plays a pivotal role in regulating epithelial cell proliferation during intestinal wound healing. Following injury, restoration of the epithelial layer requires not only cell migration but also expansion of the epithelial cell population. FAK functions as a mechanosensor that responds to changes in tissue stiffness and ECM composition, promoting cell cycle progression through the upregulation of cyclin D1 [32]. Owen et al. found that in models of colonic injury, loss of epithelial FAK results in significantly impaired proliferation, reduced cyclin D1 expression, and defective mucosal healing [9]. FAK-deficient mice exhibit increased disease severity, characterized by ulceration, disrupted crypt architecture, and extensive edema [9].

FAK supports epithelial cell survival during wound healing. Adhesion-mediated survival signaling is critical in maintaining epithelial integrity under conditions of stress and injury. FAK contributes to this process by activating pro-survival PI3K/Akt pathways and by suppressing apoptosis through multiple mechanisms, including the regulation of p53 [9]. Nuclear FAK has been shown to interact with p53 and promote its degradation, thereby inhibiting apoptosis and enhancing cell survival [9]. Loss of FAK in intestinal epithelial cells leads to increased p53 accumulation, elevated apoptosis, and impaired healing responses following inflammatory injury [9].

Lastly, FAK contributes to the restoration of epithelial barrier function, a critical endpoint of intestinal wound healing. Tight adherent junction remodeling is necessary to re-establish barrier integrity following injury. FAK activation has been shown to regulate the redistribution and assembly of junctional proteins, including claudins and E-cadherin, facilitating recovery of transepithelial resistance and barrier function. Inhibition of FAK signaling disrupts tight junction reassembly and delays barrier restoration, indicating that FAK activity is required for proper epithelial barrier repair [33,34].

## 4. NRF2 Redox Pathway in Wound Healing

Reactive oxygen species play a dual role in the gastrointestinal wound healing process that depends on their concentration, duration of production, and spatial location. Following mucosal injury, transient, spatially restricted ROS generation at the wound edge functions as a second messenger by reversibly oxidizing catalytic cysteine residues within protein tyrosine phosphatases, transiently inhibiting phosphatase activity and prolonging phosphorylation-dependent signaling pathways that regulate epithelial migration, focal adhesion turnover, proliferation, survival, and innate host defense [35,36,37]. Excessive ROS accumulation can lead to oxidative stress, tissue damage, and chronic inflammation. In contrast, sustained or excessive ROS production overwhelms endogenous antioxidant defenses, resulting in oxidative stress characterized by irreversible oxidation of proteins, lipids, and DNA, activation of pro-inflammatory signaling pathways, epithelial apoptosis, disruption of tight junction proteins, and impaired mucosal barrier repair. Consequently, successful intestinal wound healing requires maintenance of ROS within a physiological range that supports redox signaling while preventing oxidative injury. Intracellular antioxidant defense systems preserve this balance by limiting excessive ROS accumulation and maintaining redox homeostasis. Among these mechanisms, gastrointestinal epithelial cells activate several antioxidant defense systems, and the KEAP1-NRF2 signaling pathway serves as the central regulator of redox balance during wound healing.

**Figure 3:**
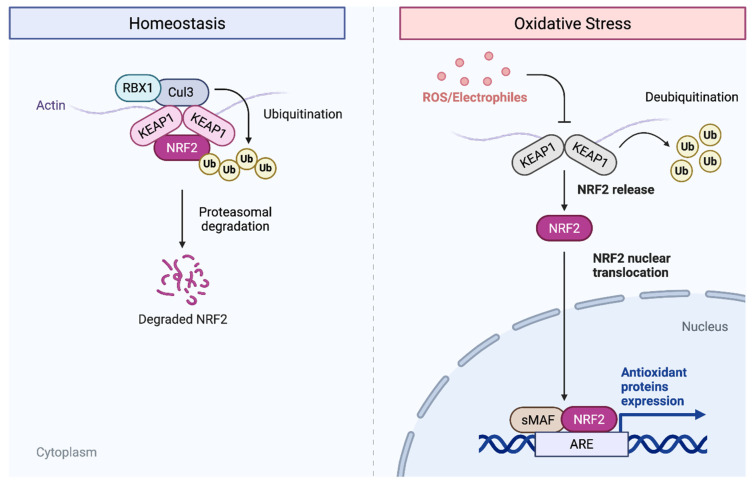
Overview of the regulation of the NRF2–KEAP1 antioxidant signaling pathway under homeostatic and oxidative stress conditions.

NRF2 is a cytoprotective transcription factor belonging to the Cap’n’Collar (CNC) family that contains a conserved basic leucine zipper (bZIP) structure. NRF2 regulates the expression of antioxidant and detoxifying enzymes in response to harmful effects of extrinsic and intrinsic insults, such as xenobiotics and oxidative stress [38]. Under normal physiological conditions, NRF2 is retained in the cytoplasm by Kelch-like ECH-associated protein 1 (KEAP1), which promotes ubiquitination and proteasomal degradation of the transcription factor (Figure 3). However, during oxidative stress, ROS and electrophiles modify cysteine residues on KEAP1, allowing NRF2 to escape degradation and translocate to the nucleus [39]. In the nucleus, NRF2 binds to antioxidant response elements (AREs) in the promoters of target genes and induces transcription of cytoprotective and detoxifying enzymes. These enzymes restore redox homeostasis and preserve epithelial viability and promote tissue repair [40,41,42]. Some of these genes include NAD(P)H: quinone oxidoreductase-1 (NQO1), glutathione reductase (GSR), glutathione-S transferase (GST), glutathione peroxidase 2 (GPX2), superoxide dismutases 1–3 (SOD1–3), glutamate-cysteine ligase catalytic subunit (GCLC), glutamate-cysteine ligase modifier subunit (GCLM), peroxiredoxins (PRX), thioredoxins (TRX), catalase (CAT), and heme oxygenase-1 (HO-1), all of which contribute to cellular antioxidant defense, thereby protecting epithelial cells from oxidative injury during wound repair [42,43,44].

Several studies have demonstrated that NRF2 contributes to the anti-inflammatory response during wound healing by inducing the expression of anti-inflammatory genes, including CD36, MARCO, and IL-17D [45,46,47]. In Nrf2-knockout mice, the anti-inflammatory effect is largely absent, resulting in an inability to properly resolve inflammatory signaling [48,49]. NRF2 also inhibits inflammation via suppression of pro-inflammatory cytokines and chemokines such as IL-6, TNF-α, IL-1β, Mip2, and MCP-1 [48,49,50,51]. This regulatory role is supported by findings showing increased production of IL-6 and IL-1β in Nrf2^−/−^ mice with dextran sulfate-induced colitis [52]. In addition, NRF2 inhibits the production of downstream IL-17 and other inflammatory factors during Th1 and Th17 responses [53]. Primary studies further demonstrate that NRF2 expression levels regulate immune cell recruitment by controlling chemokine expression including CCL2, CCL3, and CXCL2, thereby modulating neutrophil and monocyte/macrophage infiltration into inflamed tissues [54,55,56,57]. These anti-inflammatory effects are mechanistically linked to NRF2-dependent regulation of redox homeostasis, as oxidative stress is a known amplifier of pro-inflammatory signaling pathways. Reduced expression of antioxidant and phase II detoxification enzymes, including UDP-glucourosyltransferase 1A1 (UGT1A1), NQO1, HO-1, and glutathione S-transferase Mu-1 (GSTM-1), has been linked to increased severity of inflammatory diseases such as colitis in Nrf2-deficient mouse models [58,59]. Conversely, overexpression or pharmacological activation of NRF2 has been shown to reduce inflammatory damage and increase wound closure rate, demonstrating the protective role of NRF2-mediated antioxidant responses in tissue repair [60].

Experimental models of systemic injury further highlight the importance of NRF2 in regulating oxidative stress responses. For example, following burn injury, increased expression of NRF2-regulated genes such as NQO1 and GCLM has been observed in wild-type mice, whereas these responses are absent in Nrf2 knockout animals. Interestingly, the antioxidant enzyme HO-1 was increased in both wild-type and Nrf2 knockout mice following burn injury, suggesting that some cytoprotective genes may also be regulated by additional signaling pathways independent of NRF2 [48]. Consistent with these findings, a study in corneal epithelial wound healing models demonstrated that NRF2 deficiency results in significantly delayed wound closure and impaired activation of antioxidant defense systems following injury [61]. These findings indicate that NRF2 plays a crucial role in orchestrating the antioxidant transcriptional response following injury-induced oxidative stress.

## 5. Antioxidant Genes in Wound Healing

While the NRF2 signaling pathway serves as a central transcriptional regulator of antioxidant responses, its functional impact during intestinal wound healing is ultimately mediated through downstream effector enzymes that directly control intracellular redox balance. Among these, GCLC, GCLM, SOD1, CAT, and NQO1 perform distinct biochemical functions, that converge on several key biological processes required for gastrointestinal wound healing, including epithelial restitution, maintenance of barrier integrity, resolution of inflammation, and protection against oxidated epithelial injury [62].

Superoxide anions represent the primary ROS generated from molecular oxygen [63]. SOD1, also known as copper–zinc SOD (CuZnSOD), plays a primary role in the detoxification of superoxide anions by directly reacting with them to form oxygen and hydrogen peroxide (H_2_O_2_) [64]. SOD1 deficiency was found to result in severe oxidative stress with body weight loss, epithelial barrier disruption, and decreased antioxidant enzyme activities in DSS-induced Sod1-knockout mice [65]. The same study later found that rescue of SOD1 activity in the same mice significantly ameliorated enhanced DSS-induced colitis. Another study found that Sod1-knockout mice experience a redox imbalance and are prone to damage by wounding and experience a significant delay in wound healing time in older mice [66]. Beyond their antioxidant functions, SODs also exhibit immunomodulatory functions, influencing immune cell activation, cytokine production, and inflammatory responses. Abnormal SOD function has been closely associated with inflammatory diseases, where impaired ROS detoxification leads to activation of redox-sensitive inflammatory pathways and dysregulation of immune cell populations, highlighting the close interplay between oxidative stress and inflammatory signaling [65,67]. Collectively, these findings indicate that SOD1 supports gastrointestinal wound healing by limiting superoxide-mediated epithelial injury, preserving epithelial barrier integrity, and preventing excessive inflammatory responses that impair epithelial restitution. Through these actions, SOD1 creates a redox environment that favors epithelial cell survival and mucosal repair.

The generation of H_2_O_2_ by SOD1 introduces the next layer of redox regulation. H_2_O_2_ is freely diffusible, relatively long-lived and acts as a weak oxidizing agent as well as a reducing agent [68]. While moderately stable, it is the progenitor of toxic hydroxyl radicals via the Fenton reaction. CAT contributes to the secondary detoxification step of superoxide anions by converting H_2_O_2_ into water and oxygen, thereby completing the neutralization of ROS. CAT dysfunction has been associated with numerous age-related and inflammatory diseases including diabetes mellitus, hypertension, anemia, and cancer [68]. Its specific role in intestinal wound healing remains less clear but some evidence suggests it may not be essential for the skin healing process [69]. Although direct evidence linking CAT to gastrointestinal restitution remains limited, its ability to remove excess H_2_O_2_ is expected to protect epithelial cells from oxidative damage, preserve tight junction integrity, and prevent ROS-mediated inflammatory signaling that delays mucosal repair. By maintaining physiological H_2_O_2_ concentrations, CAT may also help preserve the redox signaling required for normal epithelial migration during wound healing.

In parallel with enzymatic ROS detoxification, the glutathione (GSH) system provides a central buffering mechanism that sustains intracellular redox homeostasis during wound healing. This system is directly dependent on the activity of GCLC and GCLM, which together form glutamate cysteine ligase (GCL), the rate-limiting enzyme in GSH synthesis. GSH is present in all mammalian tissues as the most abundant cytosolic non-protein thiol that defends against oxidative stress while also functioning as a key regulator of redox signaling, playing essential roles in xenobiotic detoxification and the regulation of cell proliferation, apoptosis, immune function, and fibrogenesis [70,71]. GSH acts as a cofactor for detoxifying enzymes and contributes to the regeneration of other antioxidant systems [71]. Insufficient GSH availability has been associated with impaired epithelial integrity and increased vulnerability to oxidative injury. More specifically, depletion of tissue GSH has been shown to be associated with a marked cellular degeneration of colon epithelium in ulcerative colitis [72].

Key determinants of GSH synthesis are the availability of the sulfur amino acid precursor, cysteine, and the activity of GCL. Changes in GCL activity can result from regulation at multiple levels affecting just GCLC or both GCLC and GCLM [70]. GCLC exhibits all of the catalytic activity of the isolated enzyme and feedback inhibition by GSH [73]. GCLM is enzymatically inactive but plays an important regulatory function by lowering the K_m_ of GCL for glutamate and raising the K_i_ for GSH [74]. Thus, the holoenzyme is catalytically more efficient and less subject to inhibition by GSH than GCLC [75]. However, GCLC alone does have enzymatic activity as Gclm knockout mice are viable but have markedly reduced tissue GSH levels [76]. Increased expression of these subunits during injury promotes elevated GSH production, enhancing the cell’s capacity to neutralize ROS and maintain redox equilibrium. While NRF2 is a known upstream regulator of GCLC and GCLM, recent evidence suggests that their expression may also be maintained through NRF2-independent mechanisms in a cell-type-specific manner. One study comparing neuronal and non-neuronal cells demonstrated that GSH synthesis can be differentially regulated depending on cellular context [77]. These findings indicate that antioxidant gene regulation is not universally controlled by NRF2 alone but instead operates within a broader network of signaling pathways that vary across tissue types. This observation is particularly relevant for gastrointestinal epithelial repair where multiple signaling inputs may converge to regulate redox homeostasis during wound healing. During gastrointestinal wound healing, increased GSH synthesis through GCLC and GCLM protects epithelial cells from oxidative injury, supports cell proliferation and survival, preserves tight junction proteins, and promotes restoration of the mucosal barrier. Adequate GSH availability is therefore essential for epithelial restitution while simultaneously limiting inflammatory tissue damage.

NQO1 plays a central cytoprotective role in preventing cellular oxidative damage induced by quinones through its function as a flavin-dependent antioxidant enzyme [62]. Specifically, NQO1 competes with one-electron reductases such as cytochrome P450 by catalyzing the direct two-electron reduction of quinones into more stable hydroquinones, using NADH or NADPH as electron donors [78]. Once reduced, the toxic quinones are further conjugated with GSH or glucuronic acid and excreted from the cells. This two-electron reduction is critical because it bypasses the formation of unstable semiquinone intermediates that would otherwise participate in redox cycling and generate ROS, amplifying oxidative stress [79]. By reducing quinones in this manner, NQO1 not only detoxifies reactive electrophiles but also helps preserve intracellular thiol pools and maintain overall redox homeostasis. NQO1 expression is transcriptionally regulated through the NRF2–KEAP1/ARE pathway, where NRF2 serves as a key upstream regulator [80]. Consistent with this relationship, Nrf2 knockout mice show significantly reduced expression in NQO1 and GCLM following burn-induced intestinal injury in comparison to wild-type mice [48]. Similarly, NQO1 upregulation is completely abolished in Nrf2 knockout mice following DSS-induced inflammation compared to wild-type mice [81]. These reductions reflect both lower basal expression of these NRF2-dependent antioxidant genes and a failure to mount the normal injury-induced upregulation observed in the wild-type mice. In Nqo1 knockout mice, there is spontaneous gut inflammation, which leads to more severe inflammatory symptoms in induced DSS-colitis models compared to DSS-treated wild-type mice [65]. These findings suggest that NQO1 promotes gastrointestinal wound healing by limiting oxidative damage and preserving redox homeostasis, thereby protecting epithelial cells from injury, reducing inflammatory responses, and facilitating restoration of mucosal barrier integrity following intestinal damage.

## 6. Interaction Between FAK and NRF2

Direct mechanistic studies demonstrating a complete FAK–ROS–NRF2 signaling axis during gastrointestinal wound healing are currently lacking. Several individual components of this model have been independently established in intestinal epithelial injury models, whereas others are supported by related experimental systems. Direct evidence from intestinal epithelial models demonstrated that FAK activity is directly influenced by intracellular oxidative states, as activation of FAK and its downstream signaling pathways can increase ROS production in response to noxious stimuli, while pharmacological inhibition of FAK reduces ROS levels, nitric oxide signaling, and oxidative damage through pathways including FAK/Src and PI3K/Akt [82,83]. Conversely, ROS has been shown to regulate FAK activation in intestinal epithelial cells, where bacterially induced ROS transiently inactivates redox-sensitive phosphatases such as LMW-PTP and SHP-2, resulting in increased FAK phosphorylation, FA turnover, and enhanced epithelial cell migration during restitution [84]. Together, these findings suggest a bidirectional relationship where FAK both regulates and is regulated by ROS signaling [83].

This bidirectional relationship positions ROS as a central integrator of FAK signaling during wound repair. Importantly, ROS generated during injury also serves as the primary activating signal for the KEAP1–NRF2 pathway, linking FAK-dependent redox dynamics to downstream antioxidant gene expression. In contrast, evidence linking NRF2 directly to FAK signaling is currently derived primarily from non-intestinal systems which suggests that this relationship may extend beyond indirect regulation. For example, NRF2 has been shown to transcriptionally regulate integrin expression and promote FAK-associated signaling through ROS-dependent pathways in fibroblast models, including activation of an NRF2–integrin–FAK signaling axis [85].

Taken together, these findings support a working model in which FAK and NRF2 operate within a coordinated redox-sensitive signaling network during gastrointestinal wound healing. However, several key aspects of this model remain hypothetical (Table 1). Specifically, direct evidence that FAK-generated ROS activates NRF2 in intestinal epithelial cells, or that NRF2 reciprocally regulates FAK phosphorylation and focal adhesion dynamics during epithelial restitution, has not yet been demonstrated experimentally. Rather, these interactions are inferred from studies performed independently in intestinal wound healing models and related non-intestinal experimental systems. Future mechanistic studies will therefore be required to determine whether a functional FAK–ROS–NRF2 feedback loop operates during gastrointestinal epithelial repair.

Although this review focuses on gastrointestinal wound healing, accumulating evidence suggests that redox-sensitive FAK–ROS–NRF2 signaling is unlikely to be restricted to epithelial restitution alone. Dysregulation of these pathways has also been implicated in chronic intestinal disorders, including inflammatory bowel disease (IBD), intestinal fibrosis, and colorectal cancer. In IBD, persistent ROS production and impaired NRF2 activation contribute to chronic inflammation, epithelial barrier dysfunction, and defective mucosal repair, while aberrant FAK signaling has been associated with altered epithelial migration and inflammatory responses. During fibrosis, sustained FAK activation promotes fibroblast activation, extracellular matrix deposition, and tissue remodeling, whereas NRF2 activation can attenuate oxidative stress and fibrogenic signaling. Similarly, colorectal cancers frequently exhibit constitutive activation of both FAK and NRF2, promoting tumor cell survival, proliferation, invasion, and resistance to oxidative stress. These observations suggest that the FAK–ROS–NRF2 signaling axis functions as a broader regulator of intestinal redox homeostasis whose biological outcome is highly dependent on cellular context, injury duration, and disease state. Consequently, transient activation of this pathway may facilitate epithelial repair following acute injury, whereas chronic dysregulation may contribute to persistent inflammation, pathological tissue remodeling, or tumor progression.

## 7. Conclusions

Gastrointestinal wound healing is a complex and highly regulated process that depends on the precise coordination of epithelial cell migration, proliferation, and differentiation. Central to this process is the integration of mechanical and biochemical signaling pathways that enable epithelial cells to rapidly respond to injury and restore barrier integrity. FAK signaling plays a critical role regulating these events, orchestrating FA dynamics, cytoskeletal remodeling, and survival pathways necessary for efficient epithelial restitution and tissue regeneration. Disruption of FAK function impairs these processes, underscoring its essential role in maintaining mucosal integrity.

Equally important is the regulation of oxidative stress during wound healing. The NRF2 pathway serves as a master regulator of redox homeostasis, activating a network of antioxidant and cytoprotective genes that mitigate ROS-induced damage and modulate inflammatory responses. The downstream effector enzymes, including SOD1, CAT, GCLC, GCLM, and NQO1, provide an additional layer of control by directly detoxifying ROS and maintaining intracellular antioxidant capacity. Together, these pathways highlight the intricate interplay between cellular signaling and redox regulation in intestinal repair. A deeper understanding of these mechanisms not only advances our knowledge of mucosal biology but also identifies potential therapeutic targets for conditions characterized by impaired wound healing and chronic intestinal inflammation.

Importantly, emerging evidence suggests that FAK and NRF2 may not function as independent regulators of wound healing but instead may operate within a coordinated redox-sensitive signaling network. While individual interactions between FAK, ROS, and NRF2 have been demonstrated in intestinal wound or related experimental systems, direct evidence establishing a complete FAK–ROS–NRF2 feedback loop during gastrointestinal epithelial repair is currently lacking. Thus, the model proposed here should be viewed as a framework that integrates existing findings and provides a testable hypothesis for future mechanistic studies.

## Figures and Tables

**Table 1:** Established roles of FAK, ROS, NRF2, and downstream antioxidant enzymes in intestinal wound healing, as well as proposed interactions requiring further investigation.

Biological Process	Molecules Involved	Major Mechanism	Evidence
Epithelial migration	FAK, ROS	Focal adhesion turnover	Direct
Cell survival	FAK, NRF2, GSH	PI3K/AKT, antioxidant defense	Direct
Barrier restoration	NRF2, GCLC, GCLM, SOD1, NQO1	Tight junction preservation	Direct
Inflammation resolution	NRF2, SOD1, NQO1	Reduced oxidative stress	Direct
Redox signaling	ROS, NRF2	Antioxidant response	Direct
Proposed feedback	FAK–ROS–NRF2	Coordinated signaling	Hypothetical

## Data Availability

No new data were created or analyzed in this study. Data sharing is not applicable to this article.

## Abbreviations

The following abbreviations are used in this manuscript:

AREs	Antioxidant response element
bZIP	Basic leucine zipper
CAT	Catalase
CagA	Cytotoxin-associated gene A
CNC	Cap’n’Collar
CuZnSOD	Copper–zinc SOD
ECM	Extracellular matrix
FAK	Focal adhesion kinase
FA	Focal adhesion
FAT	Focal adhesion targeting
GCL	Glutamate-cysteine ligase
GCLC	Glutamate-cysteine ligase catalytic subunit
GCLM	Glutamate-cysteine ligase modifier subunit
GPX2	Glutathione peroxidase 2
GSH	Glutathione
GSR	Glutathione reductase
GST	Glutathione-S transferase
HO-1	Heme oxygenase-1
KEAP1	Kelch-like ECH-associated protein 1
NQO1	NAD(P)H: quinone oxidoreductase-1
NRF2	Nuclear factor erythroid 2-related factor 2
NSAIDs	Nonsteroidal anti-inflammatory drugs
PRX	Peroxiredoxins
ROS	Reactive oxygen species
SOD1–3	Superoxide dismutases 1–3
TRX	Thioredoxins
UGT1A1	UDP-glucourosyltransferase 1A1
VacA	Virulence factor vacuolating cytotoxin A
Y397	Tyrosine 397
